# An Automatic Incident Detection Method for a Vehicle-to-Infrastructure Communication Environment: Case Study of Interstate 64 in Missouri

**DOI:** 10.3390/s22239197

**Published:** 2022-11-26

**Authors:** Kun Zhang, Jalil Kianfar

**Affiliations:** Department of Civil, Computer and Electrical Engineering, Saint Louis University, St. Louis, MO 63103, USA

**Keywords:** traffic incident detection, incident management, vehicle-to-infrastructure communication

## Abstract

Transportation agencies continuously and consistently work to improve the processes and systems for mitigating the impacts of roadway incidents. Such efforts include utilizing emerging technologies to reduce the detection and response time to roadway incidents. Vehicle-to-infrastructure (V2I) communication is an emerging transportation technology that enables communication between a vehicle and the infrastructure. This paper proposes an algorithm that utilizes V2I probe data to automatically detect roadway incidents. A simulation testbed was developed for a segment of Interstate 64 in St. Louis, Missouri to evaluate the performance of the V2I-based automatic incident detection algorithm. The proposed algorithm was assessed during peak and off-peak periods with various incident durations, under several market penetration rates for V2I technology, and with different spatial resolutions for incident detection. The performance of the proposed algorithm was assessed on the basis of the detection rate, time to detect, detection accuracy, and false alarm rate. The performance measures obtained for the V2I-based automatic incident detection algorithm were compared with California #7 algorithm performance measures. The California #7 algorithm is a traditional automatic incident detection algorithm that utilizes traffic sensors data, such as inductive loop detectors, to identify roadway events. The California #7 algorithm was implemented in the Interstate 64 simulation testbed. The case study results indicated that the proposed V2I-based algorithm outperformed the California #7 algorithm. The detection rate for the proposed V2I-based incident detection algorithm was 100% in market penetrations of 50%, 80%, and 100%. However, the California #7 algorithm’s detection rate was 71%.

## 1. Introduction

A component of intelligent transportation systems (ITSs), the incident management system, aims to mitigate the negative impacts of crashes. A 2007 study reported that incidents contributed to 25% of the congestion in the United States [1], and a 2017 study reported that the annual cost of congestion in the United States was USD 192 billion [2]. Additionally, traffic crashes may result in secondary crashes [3,4]. Incident management systems are developed to improve incident detection, verification, and the response process to reduce incident clearance and recovery times [5]. The reduced clearance and recovery time lead to less congestion, reduced fuel consumption and pollution, improved travel time reliability, and a reduced likelihood of secondary crashes [6]. Automatic incident detection (AID) systems, as the name implies, aim to automatically detect incidents on roadways and alert first responders, thereby reducing the response time to incidents. Expeditious detection and response could include the following benefits: (1) the prompt arrival of medical first responders could reduce the consequences of injuries; (2) first responders could set up temporary traffic control faster and thereby mitigate congestion; and (3) transportation management center (TMC) operators could notify travelers of the incident through other ITS devices, directing them to alternate routes and activating incident traffic control plans.

Traditionally, traffic incidents came to be known in only a few different ways, the most common being reports from other drivers via phone to emergency numbers, such as 112 or 911. Other methods included receiving notice by police or other officials or notice by TMC operators via closed-circuit television (CCTV) monitoring [7]. The advent of ITS technologies, such as traffic detectors, paved the way for the development of AIDs. Traffic detectors are installed on freeways to measure traffic stream characteristics, such as flow, speed, and occupancy. These data served as the traditional source of data for automatic incident detection algorithms. One common approach for detecting incidents from point-based traffic data is to compare and analyze traffic stream characteristics obtained from detectors both upstream and downstream of a roadway segment. One disadvantage of this method is that the exact location of an incident often cannot be determined [8]. If the link lengths are short or the detectors are closely spaced, then the approximate location of the incident can be known. However, a dense deployment of detectors is expensive, not only in capital costs but also in maintenance costs [9]. A potential disadvantage of point-based methods is that they may not detect an incident until the queue spills back towards downstream sensors. In addition to point-based sensor data, link travel times obtained from automatic vehicle identification (AVI) [10] and automatic vehicle location (AVL) [11] have been utilized to develop automatic incident detection methods. 

Vehicle-to-infrastructure (V2I) communication is an emerging transportation technology that enables communication between a vehicle and the infrastructure. This paper explores the application of V2I data for automatic incident detection and contributes to the field of ITS and connected vehicles in the following ways: (1) This paper contributes to the ITS body of knowledge by developing a V2I-based incident detection algorithm. To the best of the authors’ knowledge, this is the first paper that investigates vehicle snapshots collected according to the Society of Automotive Engineers’ J2735 protocol for probe data collection on freeway facilities [12]. (2) The algorithm proposed in this paper is explainable and is developed based on statistical models, unlike some machine learning-based methods that are black boxes. (3) The proposed algorithm is investigated on the basis of various market penetration rates and for various virtual segment lengths. A freeway facility is partitioned into virtual segments, and the speeds of vehicles at these virtual segments are monitored to detect incidents. A simulation testbed of Interstate 64 in St. Louis, Missouri, USA, is developed to evaluate the proposed algorithm. The proposed algorithm is evaluated during peak and off-peak periods and under various market penetration rates for V2I technology. The performance of the proposed algorithm is compared with a traditional point-based incident detection algorithm, the California #7 algorithm. 

The remainder of this paper is organized as follows. First, an overview of the V2I probe data is provided. Next, the state of the art in the simulation of V2I environments and AIDs is reviewed. Traffic incident management system architecture is briefly discussed. Then, the proposed methodology for the V2I-based incident detection is described, and performance measures for evaluating the proposed AID are defined. In the next two sections, the simulation testbed, the results obtained from the implementation of the proposed AID, and the California #7 algorithm are described. Finally, the findings of this research and directions for future research are discussed. 

### 1.1. Vehicle-to-Infrastructure (V2I) Probe Data

Vehicle probe data in a V2I environment are collected and reported to traffic management centers and other ITS subsystems. Two of the elements of V2I that facilitate the data collected are on-board units (OBUs) and roadside equipment (RSE). OBUs are installed in vehicles and record vehicle activity data at certain intervals; recorded vehicle activities are referred to as snapshots. RSE is installed at interchanges and other locations to facilitate communication between vehicles and infrastructure. When a vehicle enters an RSE coverage area, the information stored in OBUs is transmitted to the RSE. The RSE may communicate relevant information to the OBUs. 

The standards for and format of V2I probe data are established in the SAE J2735 standard [12]. Vehicle OBUs record three types of snapshots: periodic snapshots, start and stop snapshots, and event-triggered snapshots. Periodic snapshots are recorded at regular intervals. When the vehicle speed is greater than or equal to 60 mph, periodic snapshots are recorded at 20 s intervals. When the vehicle speed is less than or equal to 20 mph, snapshots are recorded at four-second intervals. For speeds between 20 mph and 60 mph, a linear approximation is used to identify the snapshot interval. Start and stop snapshots are recorded when (i) a vehicle does not move (i.e., speed = 0) for five seconds; and (ii) when there is no record of the vehicle stopping within a 15 s interval. When a stop snapshot is recorded, periodic snapshots are no longer recorded. A start snapshot is recorded when the vehicle speed exceeds 10 mph. Event-triggered snapshots are recorded when vehicle status elements change. Airbag activation is an example of an event-triggered snapshot. Regardless of snapshot type, each snapshot includes vehicle information, such as speed, position, turn signal and brake status, and airbag activation.

### 1.2. Literature Review

In this section, the state of the art in V2I simulation is reviewed, and an overview of incident detection methods is provided. Then, incident detection algorithms relevant to this paper are reviewed.

#### 1.2.1. Vehicle-to-Infrastructure Simulation

Kianfar and Edara [13] proposed a methodology to optimize the placement of RSE for travel time estimations. A simulation testbed of a downtown network in Boise, Idaho was developed using the VISSIM traffic simulation software. Data were collected following the SAE J2735 standard. A genetic-based algorithm was proposed to find the optimal deployment of RSE to maximize the RSE’s coverage index. A sensitivity analysis was conducted to evaluate the performance of the models under various market penetration rates. 

Olia et al. [14] proposed a methodology to optimize the deployment of RSE and increase the accuracy of travel time estimations. A nondominated sorting genetic algorithm was used to find the optimal location of the RSE in a 125 km long segment of a highway in the greater Toronto area. 

Lin et al. [15] introduced a compression-sensing approach to solving the data storage problems resulting from the overcollection of traffic data in a connected vehicle environment. They evaluated the effect of their data collection approach on the travel time estimation. They implemented their approach to recapture data from a safety pilot model deployment program. The case study results revealed that the compression-sensing approach reduced the travel time estimation error to 65% relative to that of conventional data collection methods. 

Chen et al. [16] proposed a new protocol to collect probe data in a V2I environment; this protocol was referred to as the R2 protocol. In the R2 protocol, a vehicle snapshot was collected only when a significant change in vehicle speed occurred. The R2 protocol was compared with the fixed 2 s, fixed 4 s, and SAE J2735 probe data collection protocols. The results indicated that the R2 protocol reduced the travel time estimation error. Additionally, fewer snapshots were collected by the R2 protocol in comparison with the fixed 2 s, fixed 4 s, and SAE J2735 protocols. 

Liu et al. [17] developed a connected vehicle simulation platform in VISSIM and OMNeT++. The simulation testbed included a freeway in Edmonton, Canada. The number of data packets lost, network latency, the reporting interval between the time when a vehicle entered the simulation network and when it reported its packet lost, and travel time estimation were used to evaluate the performance of the proposed platform. The results showed that different traffic flow patterns affected the reported interval distribution and that RSE placement affected the travel time estimation. They recommended that the RSE deployment design should consider not only the performance of data communication but also different traffic flow patterns.

#### 1.2.2. Incident Detection Algorithms

Incident detection algorithms are primarily classified into four categories: (1) pattern-based algorithms; (2) algorithms based on catastrophe theory; (3) statistical algorithms; and (4) artificial-intelligence-based algorithms [18]. Pattern-based algorithms mainly compare observed traffic parameters, such as speed, traffic volume, and occupancy, with pre-established thresholds. If the results are abnormal compared to the thresholds, an incident is identified. The California algorithm is an example of pattern-based algorithm that analyzes occupancy to detect incidents. TSC algorithm 7, TSC algorithm 8, the all-purpose incident detection (APID) algorithm, and the pattern recognition (PATREG) algorithm are all examples of pattern-based algorithms. Algorithms based on catastrophe theory detect an incident when one of the traffic parameters, such as flow, speed, and occupancy, abruptly changes while the other parameters remain unchanged. The McMaster algorithm is one such example. Statistical algorithms compare real-time traffic data obtained from roadway sensors with predicted traffic parameters for the same interval. If a discrepancy occurs between the real-time and predicted parameters, the algorithm detects an incident. The standard normal deviate (SND) algorithm and a Bayesian-based algorithm are examples of such incident detection methods. Artificial-intelligence-based algorithms learn to recognize incident patterns from historical traffic and incident data. Neural networks and fuzzy set methods have been used to develop artificial intelligence-based incident detection algorithms. AIDs are commonly assessed on the basis of three performance measures: (1) detection rate; (2) time to detect; and (3) false alarm rate. An ideal AID algorithm will achieve a high detection rate with a short period of time to detect and a low false alarm rate. However, it is not always possible to achieve an ideal balance between the detection rate, time to detect, and false alarm rate.

Hellinga and Knapp [19] proposed and evaluated the performance of three automatic incident detection algorithms (AIDs) in comparison to the McMaster algorithm. The algorithms detected incidents by evaluating changes in mean travel time, and the resolution of the proposed automatic incident detection algorithms was 20 s. The case study results indicated that the proposed algorithms outperformed the McMaster algorithm.

Deniz et al. [20] compared the performance of three categories of automatic incident detection algorithms: California #7, APID, and DES. California #7 is a derivative of the California algorithm. The all-purpose incident detection (APID) algorithm is considered a pattern-based algorithm, and the double exponential smoothing (DES) algorithm is a statistical algorithm built on time series theory. Various road conditions and traffic incident locations were used to compare the three algorithms. The results indicated that the location of incidents and road conditions, especially the traffic volume, strongly affect the performance of the algorithm.

Nathanail et al. [21] proposed a method to calibrate the California #7 incident detection algorithm that analyzes spatial differences in occupancy and spatial differences in occupancy relative to detected incidents. Threshold values for the spatial difference in occupancy and the relative spatial difference in occupancy were calibrated using the detection logic with smoothing (DELOS) algorithm. Instead of using a one-minute time interval, the DELOS algorithm utilized a three-minute average of spatial occupancy. A case study of 12 incidents indicated that the calibrated DELOS algorithm improved the detection rate from 60% to the optimum 100%.

Rossi et al. [22] proposed two incident detection algorithms that utilized fuzzy inference systems (FISs). To detect incidents, FIS1 compared the real-time traffic conditions of a roadway section with the normal traffic conditions of an upstream roadway section, and FIS2 compared the traffic conditions of a roadway section with downstream traffic conditions. The algorithms were evaluated in a simulation model of a 10 km tangent two-lane roadway. The performance of the models was assessed on the basis of the detection rate, false alarm rate, and mean time to detection. 

Asakura et al. [23] utilized GPS data obtained from commercial vehicles to detect an incident. A simulation testbed was calibrated based on commercial vehicle data and proposed algorithms that detected incidents by analyzing: (1) the link travel time; and (2) traffic shockwaves. 

Sun et al. [24] proposed an incident detection algorithm based on the set theory. The algorithm analyzed unique media access control (MAC) addresses sent to access points by the smartphones of drivers and passengers in vehicles. 

While many researchers primarily focus on incidents, such as collisions between vehicles, Yoshida et al.’s [25] research looked to identify locations of roadway damage due to natural disasters. Yoshida et al. [25] proposed a method that analyzed the three-dimensional vehicle trajectory data obtained from probe vehicles to identify the location of incidents. The three-dimensional vehicle trajectory consisted of the time (i.e., the temporal dimension) and physical location on the roadway (i.e., the physical dimension). 

Rizvi et al. [26] proposed an incident detection algorithm that applied the cell transmission model to estimate traffic flow parameters between successive traffic sensors. Macroscopic traffic flow parameters were compared with predetermined thresholds to detect incidents. 

Li et al. [27] proposed a framework to address the imbalanced incident datasets and investigate the spatial and temporal correlation between traffic stream parameters and incidents. A hybrid model that consisted of a generative adversarial network (GAN) and stacked autoencoders was proposed. Data from traffic detectors were analyzed to detect incidents. 

Evans et al. [28] proposed a random-forest-based incident detection algorithm to reduce false alarms by considering the context in the incident detection process. Holidays and sports events were examples of contexts that were factored into the incident detection process. Incidents were detected by analyzing traffic sensors’ data. 

Sun et al. [29] proposed a series of weak learners to automatically label ground truth incident data. Then the labeled ground truth data were utilized for training the machine-learning-based incident detection algorithms. Incidents were detected by analyzing the upstream and downstream traffic sensors’ data.

Iqbal et al. [30] evaluated the application of basic safety messages (BSMs) obtained from the vehicle-to-vehicle (V2V) communication environments of connected vehicles (CVs) for travel time estimations and incident detection. Regression models were used to estimate vehicle travel times. An incident was detected by comparing the prevailing traffic speeds with normal traffic speeds. The results indicated that the CV data could increase the accuracy of travel time estimations and are effective for incident detection. 

Wolfgram et al. [31] proposed an incident detection algorithm that utilized vehicle acceleration data obtained from CVs’ BSMs. The mean times to detect obtained from the case studies ranged between 20 s and 30 s. 

The review of the literature indicated that the application of V2I probe data for automatic incident detection had not been previously investigated. Iqbal et al. [30], and Wolfgram et al. [31] utilized basic safety messages (BSMs) obtained from the vehicle-to-vehicle (V2V) communication environments for automatic incident detection. However, to the best of the authors’ knowledge, this paper is the first study to propose an automatic incident detection algorithm on the basis of V2I probe data.

#### 1.2.3. Architectural Description

**Figure 1:**
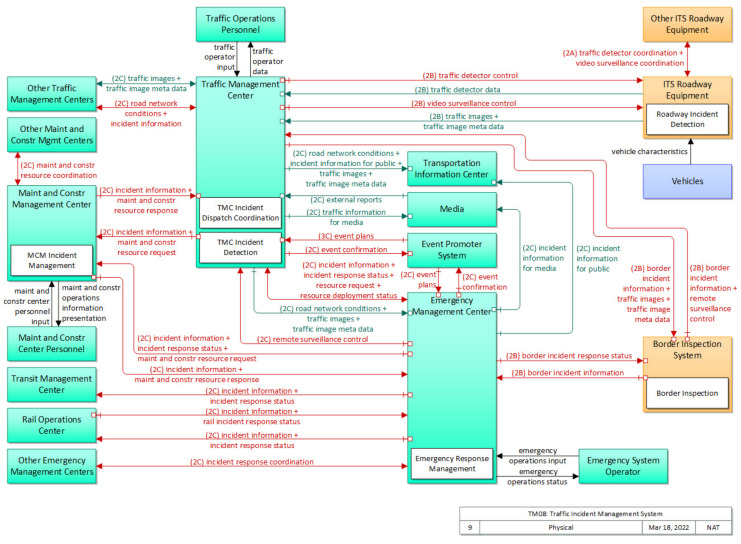
Traffic incident management system service pack: physical architecture view [35].

The incident detection algorithm proposed in this paper is a subsystem of the Advanced Traffic Management System (ATMS) and Advanced Traveler Information System (ATIS) of ITS. The fulfillment of tasks defined for various Information Technology (IT) subsystems depends on the cooperation of all the subsystems of the system [32]. A system architecture ensures interoperability between various subsystems and guarantees all the developed subsystems can be integrated into one overall solution. The V2I-based incident detection system follows the United States Department of Transportation’s National ITS Architecture [33]. The “*Architecture Reference for Cooperative and Intelligent Transportation (ARC-IT)*” consists of four views: (1) enterprise view; (2) functional view; (3) physical view; and (4) communication view [34]. The algorithm proposed in this paper falls in the traffic incident management service pack. Figure 1 demonstrates the physical view of the subsystem. Descriptions of the enterprise, functional, and physical views of the system, its goals and objectives, needs and requirements, security, standards, and system requirements are provided in [35].

In the next section, the proposed method for detecting incidents on the basis of V2I probe data is described.

## 2. Methods

The methods section is organized into three subsections. In the first subsection, the proposed algorithm for automatic incident detection using V2I probe data is explained. Next, the performance measures for assessing the automatic incident detection algorithms are defined. In the third subsection, the California #7 AID is explained. The California #7 algorithm serves as a benchmark for evaluating the results obtained from the V2I-based algorithm. California #7 utilizes point-based data (i.e., traffic sensor data) to detect incidents.

### 2.1. V2I-Based Incident Detection Method

When an incident occurs on a freeway, it often results in the temporary closure of one or more travel lanes, thus creating a temporary bottleneck. Vehicles traveling on the lanes where the incident occurred must shift their path to open lanes, and vehicles traveling on open lanes must accommodate the merging vehicles. Lane-changing maneuvers conducted by vehicles result in a temporary change in their speed and are expected to increase the measures of the dispersion of speed near the location of an incident moments after it occurrs. The algorithm proposed in this paper aims to detect incidents on a freeway by analyzing vehicle speeds reported in V2I probe snapshots. The freeway is partitioned into virtual segments, and the average speed and standard deviation of speeds in segments during detection intervals are calculated. The length of the virtual segments represents the spatial resolution of the AID, and the detection interval represents the temporal resolution of the AID. The average and standard deviation of vehicle speeds on a segment are calculated from speeds obtained from snapshots of vehicles traveling on the segment during the time interval. The average speed and standard deviation at each segment during an interval are compared with the average speeds and standard deviations reported for the same segment in the previous *δ* intervals. If the average speed and standard deviation are statistically different from those of the previous δ intervals, the algorithm identifies an incident. The AID algorithm is explained below. This algorithm is built on the work of Hellinga and Knapp [19], who utilized segment travel times obtained from automatic vehicle identification technology (AVI) through electronic toll collection (ETC) to detect incidents. Hellinga and Knapp [19] analyzed the lognormal mean and variance of the mean travel times to detect incidents and stated that mean travel times on a freeway section are lognormally distributed.

The standard deviation of speeds of vehicles equipped with connected vehicle technology that are traveling on a segment is calculated according to (1):

(1) $$\sigma_{i,j}=\sqrt{\frac{\sum_{k=1}^{n_{i,j}}\sum_{l=1}^{c_{i,j,k}}{\left(s_{i,j,k,l}-{\bar{S}}_{i,j}\right)}^{2}}{\sum_{k=1}^{n_{i,j}}c_{i,j,k}}}$$

(2) $${\bar{S}}_{i,j}=\sum_{k=1}^{n_{i,j}}s_{i,j,k,l}/\sum_{k=1}^{n_{i,j}}c_{i,j,k}$$

where 

$\sigma_{i,j}$ = standard deviation of speed snapshots of all vehicles in segment *j* at time interval *i;*

$n_{i,j}$ = number of vehicles traveling on segment *j* at interval *i;*

$c_{i,j,k}$ = number of snapshots reported for vehicle *k* in segment *j* at time interval *i;*

$s_{i,j,k,l}$ = speed in the *l*th snapshot reported by vehicle *k* while traveling on segment *j* at time interval *i;*

${\bar{S}}_{i,j}$ = average speed of all reported snapshots in segment *j* at interval *i.*

The mean of the standard deviation of speeds during the previous δ intervals is calculated according to (3):

(3) $${\bar{\sigma }}_{\delta ,j}=\frac{1}{\delta }\sum_{t=i}^{i-n_{\delta }}\sigma_{t,j}$$

where 

${\bar{\sigma }}_{\delta ,j}$ = mean of standard deviations of speed $\sigma_{t,j}$ in the comparison window for segment *j;*

δ = number of intervals in the comparison window.

The variance of the standard deviation of speed ($var_{\delta ,j}$) for segment *j* in the previous δ intervals is calculated according to (4):

(4) $$var_{\delta ,j}=\sqrt{\frac{1}{n_{\delta }-1}\sum_{t=i}^{i-\delta }{\left(\sigma_{t,j}-{\bar{\sigma }}_{\delta ,j}\right)}^{2}}$$

The following three steps are followed to detect incidents in a virtual segment:

Step 1: The lognormal mean (5) and lognormal variance (6) of the standard deviation of speed for previous δ intervals are calculated to determine the threshold values for the detection of incidents. The incident threshold values are calculated according to (7).

(5) $$\mu_{j}=\ln({\bar{\sigma }}_{\delta ,j})-0.5\tau_{\delta ,j}{}^{2}$$

(6) $$\tau_{\delta ,j}{}^{2}=\ln(1+\frac{var_{\delta ,j}}{{\bar{\sigma }}_{\delta ,j}{}^{2}})$$

(7) $$CW_{i+1}=e^{\left(\mu_{j}+Z\tau_{\delta ,j}\right)}$$

where

$\mu_{j}\;$= lognormal mean of ${\bar{\sigma }}_{\delta ,j}$;

${\bar{\sigma }}_{\delta ,j}\;$= mean of standard deviations of speed $\sigma_{\delta ,j}$ in the comparison window for segment *j* as previously defined in (3);

$\tau_{\delta }$ = lognormal variance of $var_{\delta ,j}$;

Z = Z-value for the confidence level;

$CW_{i+1,j}$ = threshold for the standard deviation of speed segment *j* for current time interval *i* + 1.

Step 2: The average speed for the previous $n_{\delta }$ intervals is calculated according to (8):

(8) $$\rho_{\delta }=\frac{1}{n_{\delta }}\sum_{t=i}^{i-\delta }{\bar{S}}_{t,j}$$

where

$\rho_{\delta }$ = average speed in the comparison window (i.e., the previous $n_{\delta }$ intervals).

Step 3: If the standard deviation of speed for the current interval is greater than the corresponding upper limit ($CW_{i+1}$) and the current speed is smaller than the average speed for the reference sample ($\rho_{\delta }$), an incident has occurred in segment *j* in the time interval *i* + 1. In the next subsection, performance measures for evaluating the developed algorithm are described. 

### 2.2. Incident Detection Performance Measures

The performance of the AID algorithms was assessed based on the detection rate, time to detect, detection accuracy, and false alarm rate. These performance measures are defined as follows.

The detection rate (DR) is the number of incidents correctly detected by the algorithm divided by the total number of incident cases. Time to detect (TTD), as the name implies, is the time interval between the moment an incident occurred and the moment when the incident was detected by the algorithm. Detection accuracy and the false alarm rate are calculated based on true positive, false positive, true negative, and false negative values. True positive (*TP*) is the number of instances in which an incident occurred and was detected by the algorithm. False positive (*FP*) is the number of instances in which no incident occurred on the freeway, but the algorithm incorrectly detected an incident. True negative (*TN*) is the number of instances in which no incident occurred on the freeway, and no incident was detected by the algorithm. False negative (*FN*) is the number of instances in which an incident occurred on the freeway but was not detected by the algorithm. Detection accuracy (*DA*) is the number of cases correctly detected by the algorithm divided by the total number of cases, as shown in Equation (9).

(9) $$DA=\frac{TP+TN}{TP+TN+FP+FN}\times 100$$

The false alarm rate (*FAR*) is the ratio of false positives to the total number of instances, as shown in Equation (10).

(10) $$FAR=\frac{FP}{TP+TN+FP+FN}\times 100$$

*DA* ranges between 0 and 100, with higher values indicating better model performance. *FA* ranges between 0 and 100, with smaller values indicating better model performance. An ideal AID will maintain balance among *DA*, *FAR*, and TTD and will result in high *DA*, low *FAR*, and low TTD. The next subsection describes the benchmark algorithm for automatic incident detection. 

### 2.3. Point-Based Incident Detection Method

**Figure 2:**
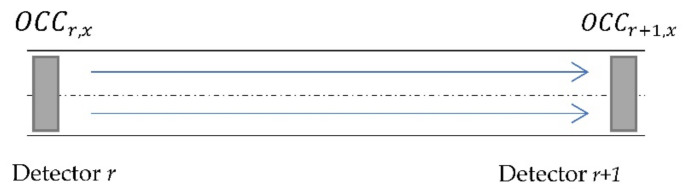
Sample section of a freeway for California #7 AID algorithm.

The California #7 algorithm was used as a benchmark to evaluate the performance of the proposed V2I-based AID algorithm. Figure 2 shows an example of a section of a freeway with a detector upstream of the section and a detector downstream of the section. The California #7 algorithm analyzes occupancy (*OCC*) measured at the upstream detector (*r*) at time interval (*x*) and occupancy measured at the downstream detector (*r* + 1) at time interval (*x*) to detect an incident that has occurred between upstream and downstream detectors. The California #7 algorithm computes the difference in occupancy ($OCCDF_{r,x}$) and the relative difference in occupancy ($OCCRDF_{r,x}$) for segment r at a time interval x. The algorithm steps are outlined below:

Step 1: Calculate *OCCDF* between upstream and downstream detectors at time interval x:

(11) $$OCCDF_{r,x}=OCC_{r,x}-OCC_{r+1,x}$$

Step 2: Calculate *OCCRDF*:

(12) $$OCCRDF_{r,x}=\left(OCC_{r,x}-OCC_{r+1,x}\right)/OCC_{r,x}$$

Step 3: Calculate the difference between downstream occupancy *DOCCDF* during two successive detection intervals:

(13) $$DOCCDF_{r,x}=OCC_{r+1,x}-OCC_{r+1,x+1}$$

Step 4:

- If $OCCDF_{r,x}$ > *T1*, an incident occurred but is not detected by the algorithm;
- If $OCCDF_{r,x}$ > *T1* and $OCCRDF_{r,x}$ > *T2*, an incident occurred and is detected by the algorithms;
- If $OCCDF_{r,x}$ > *T1*, $OCCRDF_{r,x}$ > *T2*, and $DOCCDF_{r,x}$ > *T3*, an incident that has already been detected continues. Otherwise, no incident occurred.

*T1* is the threshold for maximum *OCCDF* during normal traffic conditions; *T2* is the maximum value of the difference in *OCCRDF* during normal traffic conditions; and *T3* is the maximum *DOCCDF* during normal traffic conditions. Nathanail et al. [21] outlined the process for calibration of *T1*, *T2*, and *T3* thresholds.

## 3. Results

### 3.1. Simulation Testbed

A 2.13 km (7000 ft) section of Interstate 64 in St. Louis, Missouri, USA was selected as the case study location. The freeway section was coded in the VISSIM traffic simulation software, and the SAE J2735 Dedicated Short Range Communications (DSRC) protocol for probe data collection was coded in MATLAB. The VISSIM COM interface was used to connect the traffic simulation models with the V2I probe data collection module in MATLAB. 

**Figure 3:**
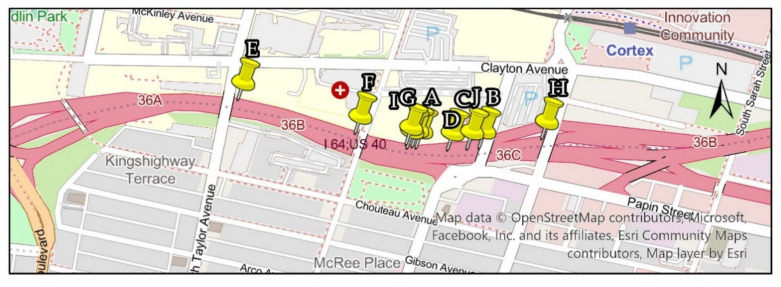
Boundaries of simulation testbed and location of incidents.

Ten real-world incidents that occurred on Interstate 64 were identified in the traffic management center data logs and were simulated in the model. The duration and coordinates of the incidents were obtained from the incident logs, and traffic volumes before, during, and after an incident were retrieved from traffic sensors located upstream of the incident location. Incidents were selected to represent diverse traffic patterns. The selected incidents occurred during peak and off-peak periods and during summer, fall, and winter. Table 1 provides the date, start time, and duration of the incidents. The location of the incidents is illustrated in Figure 3. Each event was simulated for 2 h and 30 min. The initial 30 min of the simulation was considered a warm-up period, and no data were collected during this period. The ten incidents in the case study were selected to represent various roadway and environmental factors. These factors included the duration of incident clearance times and severities, peak and off-peak periods, seasons, roadway geometry, and driver behaviors. It is worth to mention the size of the case study is a limitation of the work presented in this paper. In the future, it will be beneficial to investigate the V2I-based algorithm with a larger set of real-world incidents and with different types of roadway facilities.

### 3.2. Performance of V2I-Based AID Algorithm

**Figure 4:**
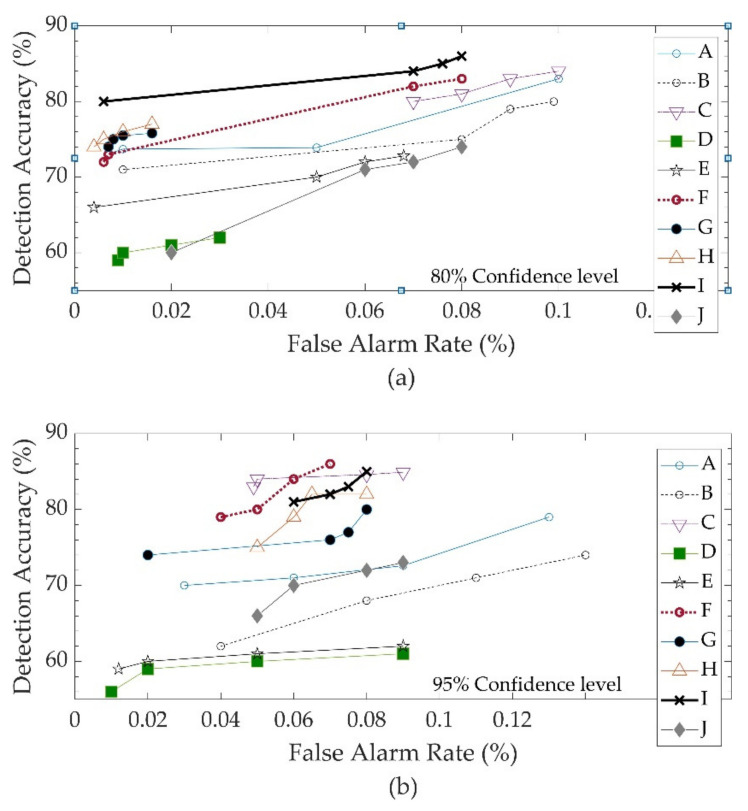
Relation between DA and FAR at 500 ft virtual segments and 100% market penetration: (**a**) 80% confidence level; (**b**) 95% confidence level.

Traffic incidents were simulated in the testbed, and the V2I-based automatic incident detection algorithm was evaluated under various V2I market penetration rates, two different virtual segment lengths, and two different detection levels of confidence. The performance of the AID was investigated under four different levels of market penetration for OBU-equipped vehicles: 20%, 50%, 80%, and 100%. With regards to the length of virtual segments, the performance of the algorithm was investigated for 500 ft and 1000 ft virtual segments. Furthermore, the performance of the model with levels of confidence (Z) of 80% and 95% was investigated. The DA, DR, FAR, and TTD were reported for each simulation scenario. Figure 4a,b illustrates the relation between the FAR and DA for virtual segments of 500 ft. Figure 4a shows the results for a confidence level of 80%, and Figure 4b displays the FAR and DA for a confidence level 95%. There are four data markers on each data series line on these figures. The first marker from the left corresponds to a 20% market penetration rate, the second marker corresponds to a 50% market penetration rate, the third marker corresponds to a 80% market penetration rate, and the fourth marker corresponds to a 100% market penetration rate. With a confidence level of 80%, the DA varied between 55% and 80%, and the maximum FAR of the algorithm was 18%. With a confidence level of 95%, the maximum FAR improved to 12%, and the DA improved with values ranging between 60% and 90%. As expected, the algorithm with a confidence level of 95% outperformed the algorithm with a confidence level of 80%. As a result, a confidence level of 95% was selected as an acceptable value for the confidence level, and a sensitivity analysis was conducted with respect to the market penetration and length of virtual segments. 

**Figure 5:**
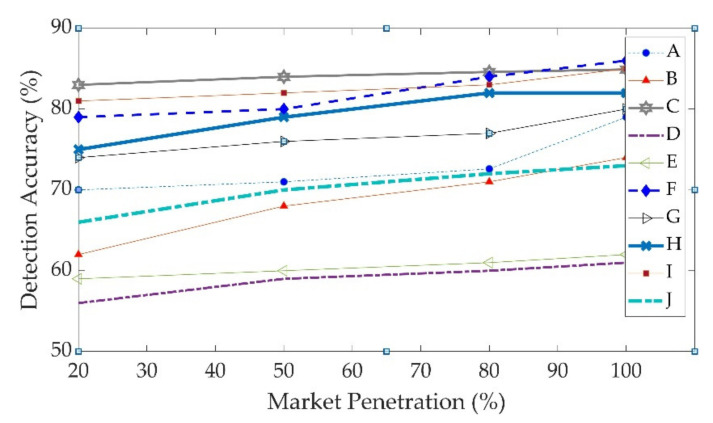
Relation between market penetration and DA for incidents A to I.

Table 2 reports the DA, FAR, and TTD for the case study incidents. These performance measures are reported for market penetrations of 20%, 50%, 80%, and 100%, and for virtual sections of 500 ft and 1000 ft. The performance measures reported in Table 2 reveal several trends:

- In general, when market penetration increased, the DA and FAR improved. This is not surprising because when the market penetration increases, the quality of probe data improves as more snapshots are reported to the V2I AID algorithm;
- The FAR and DA were not correlated with the length of the virtual segment. However, the TTD decreased from 1.7 min to 1.3 min when the virtual segment length was reduced from 1000 ft to 500 ft.
- It is worth mentioning that as the market penetration increased, the FAR increased. This is because an increase in the number of probe snapshots increased the number of FPs. However, the maximum observed FAR was less than 12%, which is considered desirable;
- Increasing the market penetration from 20% to 50% resulted in meaningful improvements in the DA; however, increasing market penetration from 50% to 100% did not have a significant impact on the DA. This trend is illustrated in Figure 5;
- When the market penetration increased from 20% to 50%, the TTD improved. However, when the market penetration increased from 50% to 80%, no meaningful improvements in the TTD were observed;

### 3.3. V2I-Based AID Algorithm vs. California #7 Algorithm

The performance of the proposed V2I-based AID algorithm was compared with the performance of the California #7 algorithm. First, the California #7 algorithm was modeled in the simulation testbed and was calibrated for Interstate 64. Inductive loop detectors were in 500 ft intervals in the testbed. Ground truth data collected by the Missouri department of transportation traffic sensors were used to simulate traffic on 32 workdays on which no incidents occurred on Interstate 64. The data collected by simulation testbed inductive loop detectors were used to determine the thresholds of the California #7 algorithm according to the process described by Nathanail et al. [21]. The T1 threshold was 0.18%, T2 threshold was 0.27%, and T3 threshold was 0.5%. 

Next, incidents A to J were modeled in the testbed, and the V2I-based AID algorithm and California #7 algorithm were simulated. The V2I-based AID algorithm was modeled with virtual segments that were 500 ft long and with market penetration rates of 50%, 80%, and 100%. The TTD, FAR, and DR obtained for both algorithms are reported in Table 3. 

For all three market penetration scenarios, the V2I-based AID algorithm’s DR was 100%, and the V2I-based AID algorithm consistently outperformed the California #7 algorithm. With regards to the FAR, the V2I-based AID algorithm outperformed the California #7 algorithm at the market penetration rates of 80% and 100%. However, the California #7 algorithm had a lower FAR than the V2I-based AID algorithm at 50% market penetration. Similarly, the TTD of the V2I-based AID algorithm at 50% market penetration was comparable to that of the California #7 algorithm, and when the market penetration was increased to 50% and 100%, the TTD improved by 39% and 50%, respectively.

## 4. Discussion

Traffic incidents are a source of non-recurrent congestion and may result in secondary crashes. The prompt detection of roadway incidents enables transportation agencies to employ a spectrum of strategies to mitigate the impacts of incidents and prevent additional crashes. AIDs enable transportation agencies to detect incidents on the roadway and implement the appropriate response automatically. Improving the performance of AIDs has an overall positive impact on incident clearance and the recovery process and reduces the environmental and economic costs of incidents. AIDs commonly utilize point sensors data, GPS and AVL data, and CCTV video feeds to detect incidents. This paper investigated the application of emerging sources of data—vehicle snapshots generated by OBUs—in the V2I environment for automatic incident detection. A novel V2I-based incident detection algorithm was proposed, and its performance was compared with a conventional AID algorithm. 

A simulation testbed of a segment of Interstate 64 in St. Louis, Missouri was developed, and real-world incidents were modeled to evaluate the performance of the proposed incident algorithm. The detection rate (DR), detection accuracy (DA), false alarm rate (FAR), and time to detect (TTD) were used to evaluate the proposed algorithm. A sensitivity analysis was conducted to assess the performance of the proposed algorithm under two confidence levels of 80% and 95%, four market penetrations of 20%, 50%, 80%, and 100%, and two different virtual freeway detection segments lengths of 500 ft and 1000 ft. The performance of the algorithm significantly improved when the market penetration was increased from 20% to 50%; however, the same level of benefit was not achieved when the market penetration was increased from 50% to 80% and 100%. The performance of the proposed V2I-based AID algorithm was compared with that of the California #7 algorithm. With market penetrations of 80% and 100%, the V2I-based AID algorithm significantly outperformed the California #7 algorithm.

The V2I-based incident detection method proposed in this paper has several advantages and disadvantages. These strengths and weaknesses are discussed in the following two subsections.

### 4.1. Strengths of the Algorithm

Unlike machine-learning-based incident detection algorithms, which are often black boxes, the algorithm proposed in this paper follows statistical principles. This statistics-based approach makes the algorithm explainable and straightforward to implement for practitioners. The algorithm does not rely on propriety codes and does not require the extensive training needed for machine learning methods. Another strength of the algorithm is that the incident detection segments are defined virtually. In the conventional incident detection methods, such as the California #7 algorithm, the detection segments are constrained between the physical location of the two consecutive sensors on the road. As such, additional sensors should be installed on the road to shorten the length of the detection segments. However, in the V2I-based approach, the detection segments are defined virtually, and their lengths can be adjusted from the TMC. This flexibility would allow transportation operators to adjust the lengths of the detection segments throughout the day based on the market penetration or volume for the vehicles equipped with OBUs. For example, during overnight hours, when traffic volume is low, the TMC operators can adjust the length of detection segments to be longer to improve the algorithm’s performance. Another potential advantage of the algorithm proposed in this paper is its ability to identify which lanes are blocked because of an incident. The SAE J2735 messages include the coordinates of vehicles. After an incident is detected by the algorithm, it is possible to analyze the latitudes and longitudes received from vehicles to determine where the lane changes maneuvers are occurring and from which lanes’ latitudes and longitudes are not received. This information could determine how many lanes are blocked as a result of an incident. It is worth mentioning this feature was not investigated in this paper and is suggested as a research topic for future studies. It should be noted that conventional incident detection algorithms are not able to determine which lanes are blocked due to an incident.

### 4.2. Weaknesses of the Algorithm

The V2I-based incident detection method is not without disadvantages. For example, as illustrated in Table 3, the method requires a market penetration rate of at least 80% in order to outperform the conventional method. While the conventional methods only rely on the traffic sensors data that are owned by the Departments of Transportation (DOTs), the V2I-based method depends on consumer data. In other words, the DOTs do not have complete control over the data flow. False positives are a concern for incident detection algorithms. To address this concern, the TMC operators utilize multiple sources to verify an incident. These sources include travelers’ phone calls to emergency numbers, highway patrol reports, and CCTV cameras. However, V2I-based incident detection methods may be subject to additional factors for false incident detection. One such factor relates to cyber security attacks and involves malicious actors. To address the incident verification problem, Ahmed et al. [36] proposed a method based on blockchain technology. They proposed a privacy-aware mechanism to enable vehicles from non-trusted domains to review other vehicle messages’ authenticity and reliability. Then, they proposed a blockchain-enabled framework to ensure the trustworthiness of the messages transmitted by RSUs. 

## 5. Conclusions

This paper investigated the application of an emerging source of data—vehicle snapshots generated by OBUs in the V2I environment—for automatic incident detection. A novel V2I-based incident detection algorithm was proposed, and its performance was compared with that of a conventional AID algorithm. Virtual incident detection segments were defined, and vehicle snapshots received from vehicles were analyzed to detect incidents. An incident was detected by comparing the standard deviation of speeds with the mean of the standard deviation of vehicle speeds in the previous detection intervals. The proposed algorithm was evaluated in a case study of a section of Interstate 64 in St. Louis, Missouri, USA, and the incident detection performance metrics were compared with the California #7 algorithm. The V2I-based algorithm significantly outperformed the California #7 algorithm at market penetrations of 80% or higher.

In future research, it is recommended to evaluate V2I-based incident detection algorithms on arterial streets and other interrupted-flow facilities. It is also recommended to evaluate the V2I-based technologies against algorithms that utilize AVL and AVI data. Finally, it is recommended to develop AIDs that fuse V2I sensors’ data with conventional data sources to detect roadway incidents.

## Figures and Tables

**Table 1:** Incidents modeled in the simulation testbed.

Incident ID	Incident Date	Incident Start Time	Incident Duration (min)
A	17 January 2015	9:08 AM	24
B	18 December 2015	10:16 AM	46
C	21 December 2015	11:31 AM	35
D	20 January 2016	5:37 AM	64
E	20 January 2016	9:03 AM	65
F	4 June 2016	9:40 AM	25
G	4 July 2016	9:02 AM	22
H	29 July 2016	7:57 PM	22
I	19 September 2016	4:25 PM	11
J	6 October 2016	7:59 AM	38

**Table 2:** Sensitivity analysis of the performance of V2I-based AID.

Incident	Market Penetration (%)	DA (%)		FAR (%)		TTD (min)	
		1000 ft Segment	500 ft Segment	1000 ft Segment	500 ft Segment	1000 ft Segment	500 ft Segment
A	20	70	73.1	3	0.7	7	10
	50	71	73.7	6	1	3	2
	80	72.6	73.9	9	5	2	1
	100	79	83	13	10	0	0
B	20	62	71	4	1	5	3
	50	68	75	8	8	3	2
	80	71	79	11	9	1	0
	100	74	80	14	9.9	0	2
C	20	83	80	4.9	7	5	4
	50	84	81	5	8	2	2
	80	84.6	83	8	9	2	1
	100	84.9	84	9	10	1	1
D	20	56	59	1	0.9	4	5
	50	59	60	2	1	3	0
	80	60	61	5	2	1	1
	100	61	62	9	3	0	1
E	20	59	66	1.2	0.4	8	5
	50	60	70	2	5	4	3
	80	61	72	5	6	1	2
	100	62	72.8	9	6.8	1	1
F	20	79	72	4	0.6	8	10
	50	80	73	5	0.7	7	2
	80	84	82	6	7	4	1
	100	86	83	7	8	2	1
G	20	74	74	2	0.7	6	8
	50	76	75	7	0.8	3	2
	80	77	75.5	8	1	3	2
	100	80	75.8	7	1.6	2	1
H	20	75	74	5	0.4	9	7
	50	79	75	6	0.6	5	4
	80	82	76	6.5	1	2	0
	100	82	77	8	1.6	2	0
I	20	81	80	6	0.6	4	3
	50	82	84	7	7	2	2
	80	83	85	75	7.6	1	2
	100	85	86	8	8	1	1
J	20	66	60	5	2	7	6
	50	70	71	6	6	3	0
	80	72	72	8	7	2	1
	100	73	74	9	8	1	1

**Table 3:** Performance measures for V2I-based AID and California #7 algorithms.

Algorithm	Market Penetration MP (%)	Detection Rate DR (%)	False Alarm Rate FAR (%)	Time-To-Detect TTD (min)
V2I-based AID	50	100	38.3	1.9
	80	100	5.5	1.1
	100	100	6.7	0.9
California #7	100	71	21.0	1.8

## Data Availability

The data presented in this study are available on request from the corresponding author.
